# Scoping Review of the Environmental and Human Health Effects of Rural Alaska Landfills

**DOI:** 10.3390/ijerph23010045

**Published:** 2025-12-30

**Authors:** Carlye Chaney, Anita Moore-Nall, Chad Albert, Catherine Beebe, Britta Bierwagen, Michelle Davis, Alice Demoski, Angel Ip, Page Jordan, Sylvia S. Lee, Edda Mutter, Lauren Oliver, Nichol Rallo, Kate Schofield, Johnee Seetot, Anastasia Shugak, Angalgaq Tom, Martha Turner, Lynn Zender

**Affiliations:** 1 Department of Biology, Washington University in St. Louis, St. Louis, MO 63110, USA; 2 Department of Anthropology, University of Missouri, Columbia, MO 65211, USA; 3 Department of Native American Studies, Montana State University, Bozeman, MT 59715, USA; 4 Northway Village, Northway, AK 99764, USA; 5 Native Village of Kwinhagak, Quinhagak, AK 99655, USA; 6 Office of Research and Development, U.S. Environmental Protection Agency, Washington, DC 20460, USA; 7 Region 10, U.S. Environmental Protection Agency, Anchorage, AK 99513, USA; 8 Nulato Village, Nulato, AK 99765, USA; 9 Region 10, U.S. Environmental Protection Agency, Seattle, WA 98101, USA; ip.angel@epa.gov; 10 Office of Research and Development, U.S. Environmental Protection Agency, Cincinnati, OH 45268, USA; 11 Yukon River Inter-Tribal Watershed Council, Anchorage, AK 99501, USA; emutter@yritwc.org; 12 Native Village of Brevig Mission, Brevig Mission, AK 85039, USA; 13 Alutiiq Tribe of Old Harbor, Old Harbor, AK 99643, USA; 14 Zender Environmental Health and Research Group, Anchorage, AK 99501, USA; lzender@zendergroup.org

**Keywords:** landfill, Alaska native, solid waste, rural, contaminants, environmental health, subsistence

## Abstract

**Highlights:**

**Public health relevance—How does this work relate to a public health issue?**

- This work reviews articles related to landfills in rural Alaska communities and may guide future research related to gaps identified in this review.
- Unlined landfills lacking leachate collection systems may impact public health.

**Public health significance—Why is this work of significance to public health?**

- This work identifies several potential disease transmission and chemical exposure pathways associated with such landfills, which are primarily found in rural and tribal communities already facing significant health disparities.
- If harvested, birds, fish, other animals and plants living, growing, or feeding by open surface area landfills could present public health implications for Indigenous and other hunting populations via ingestion or contact with waste pathogens or chemicals.

**Public health implications—What are the key implications or messages for practitioners, policy makers and/or researchers in public health?**

- This review paper elucidates the need to continue to assess landfill contaminant transport, exposure pathways and risks and the unique challenges of solid waste management in rural Alaska and other Arctic environments.
- Future research directions regarding the risk to subsistence resources and the associated health implications for Alaska Native and other Arctic subsistence-based cultures should prioritize community-based, co-produced research that integrates Indigenous Knowledge with Western science. This approach is crucial for addressing the existing health disparities and unique environmental exposures faced by these communities.

**Abstract:**

Landfill contaminants pose significant risks to environmental and human health, particularly in rural Alaska. These communities are predominantly Alaska Native and face unique challenges in solid waste management due to geography, climate, and limited infrastructure. This scoping review assessed published research on the impacts of landfill contaminants in the Arctic (Aim 1) and Alaska specifically (Aim 2). Seventy-one studies met the inclusion criteria, all of which were used to develop a conceptual model of contaminant transport pathways. Thirty-nine studies included Alaska-specific research: thirty-three focused on environmental impacts, and six addressed human health (e.g., birth outcomes, cancer). Key topics included waste burning, heat generation, carbon release, leachate characterization, and water or sediment contamination. Evidence specific to Alaska suggested landfill leachate may contaminate surface water and groundwater, and that microbes can migrate beyond the landfill site boundaries in communities using honeybuckets (plastic bag-lined buckets that collect human waste). Landfill contaminants also impacted wildlife through consumption of garbage, which may have human health implications for subsistence-based communities. Major research gaps remain in understanding individual-level exposures, the effects of emerging contaminants, and the mechanisms of contaminant transport pathways. Further research designed for causal inference is needed to support improvements to public and environmental health.

## 1. Introduction

Indigenous peoples often experience greater exposure to environmental contaminants [1]. These issues are frequently exacerbated for rural communities located in extreme climates, where logistical, structural, and cultural barriers tend to create conditions that can lead to environmental management challenges [2,3,4]. In particular, Alaska Native communities experience unique environmental challenges due to Alaska’s remoteness, an Arctic climate with extreme temperatures and precipitation, historical interactions with government entities, the politics of government-to-government relations with the U.S., and westernized resource management practices that threaten culturally critical natural resources. These challenges coalesce around many issues, such as food and water security, economic opportunities, and housing conditions. However, solid waste infrastructure is a particularly urgent issue in rural Alaska Native communities, given its consequences for both environmental and human health.

Solid waste refers to any garbage, refuse, sludge, or other discarded material from communities, industry, commercial activities, mining, or agriculture [5]. Varied activities are necessary to dispose of this waste, but one primary method is the operation of municipal landfills. In Alaska, municipal landfills are divided into three classes based on several criteria, including the amount of daily solid waste they receive: Class I (>20 tons of solid waste/day), Class II (5–20 tons of solid waste/day), and Class III (<5 tons of solid waste/day) [6]. Effective landfill operation is essential for both ecological health and public health [7,8,9,10]. However, systematic reviews of the literature have largely failed to observe consistent relationships between landfill proximity, environmental degradation, and human health outcomes [11,12,13]. Some of this inconsistency is likely due to variation in geography, municipal landfill management, and human activities, which contribute to differences in contamination risk and exposure across sites. These inconsistent findings emphasize the importance of understanding the site-specific context and highlight the need for a local approach to assess landfill contaminant risk.

The management of municipal landfills is distinctly challenging in rural Alaska due to its geography, limited physical infrastructure, and climate variability and extremes. Alaska is the largest U.S. state (1,717,856 km^2^), with a highly seasonal climate that experiences extremes in both temperature (−62.2 °C/−80 °F to 37.8 °C/100.4) and precipitation (<10 inches/year to >200 inches/year), crossing multiple climate zones [14]. Over half of the state’s population lives in Anchorage (population = 286,075), Fairbanks (31,856), or Juneau (31,555) [15]. Regional hub cities, such as Nome and Bethel, have populations numbering in the few thousands. These hub cities provide primary health, commercial, institutional, sport, and jet services to ‘outlying,’ rural communities. The majority of these rural communities contain fewer than 1000 residents and are predominantly Alaska Native, particularly outside of Southeast Alaska. The road system connects only 14% of the state’s communities, with the greatest concentration in the state’s south and central areas. The remaining 82% of communities are inaccessible by road, including 251 that are only accessible by air [16].

**Figure 1:**
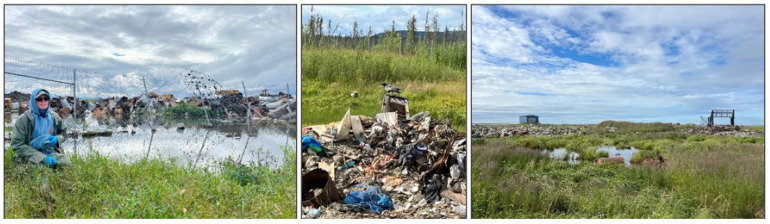
Example photos of three Class III landfills from site visits in August 2023. Left to right: E. Mutter is seated outside of the Sitaisaq (Native Village of Brevig Mission) landfill, an above-ground landfill. Middle is the Noolaaghe Doh (Nulato) landfill, a tundra pond site with a bird sitting on uncovered waste. Right is the Kuinerraq (Native Village of Kwinhagak), landfill, another above-ground site. The burn box is to the right in the right photo. Water ponding and uncovered waste are present in each of these landfills (photo credit A. Moore-Nall).

Community remoteness and extreme seasonal variation combined with other factors, such as the increasing vulnerability of permafrost to climate-related shifts, makes the design, construction, and maintenance of built infrastructure costly. These expenses have resulted in limited infrastructure even in hub cities. Given these unique circumstances, landfills in Alaska are exempt from the Solid Waste Disposal Act requirements that would be “too costly or unfeasible in remote settings” [17]. As a result, each rural community depends on Class III landfills that are unlined and lack a leachate collection system, sometimes referred to as “open dumps.” The most common Class III landfill designs are landfills located above ground, landfills contained within trenches, and landfills located in tundra ponds (see Figure 1) [18]. Waste is typically hauled to these unlined landfills by a landfill operator or residents using a truck, snow machine, or four-wheeler. Waste is deposited in the landfill and regularly covered with a thin layer of soil, gravel, or other local material. Many communities also have burn boxes—a device in which combustible solid waste can be burned at low temperature (~300 °C) to reduce the volume of solid waste.

In total, there are 184 Class III village landfills [19]. The average per capita waste production in rural Alaska is around 0.85 kg/person/day (1.88 lbs/person/day) [20]. As an example, the per capita waste production in Upper and Lower Kalskag is 0.48 kg/person/day (1.06 lbs/person/day), with the waste stream composed of paper products (19.2%), food waste (14%), “other” trash (13%), bathroom/medical waste (12.6%), diapers (12.4%), plastics (10%), cardboard (6.3%), metal products (5.3%), aluminum cans (3.4%), glass (2.5%), and newspaper (1.2%) [21]. Class III landfills are not currently designed to address large appliances, recyclables, or hazardous household waste, including batteries and electronics. Instead, these materials must be backhauled. Backhaul is a logistically and financially challenging process involving the removal of solid waste from remote communities to a different, often distant disposal location or recycling processor. This requires multiple flights, which is extremely costly and limits what can be sent out of communities, or barge service, which restricts when waste can be removed (e.g., 1 to 3 times during summer).

Further, sewage waste infrastructure contributes to health concerns related to waste disposal. Over 3300 rural Alaska homes lack piped water for flushing toilets [22], while many others rely on aging systems that can experience sewer service breaks, particularly in winter when pipes freeze. These homes must rely on “honeybuckets,” or dry toilets, which are containers lined with plastic bags to collect human waste. In at least 30% of rural communities, some honeybucket waste is comingled with solid waste, increasing the risk of disease transmission [23,24].

Alaska Native communities handle the challenges of landfill management in this extreme environment incredibly well, demonstrating resourcefulness and resilience [25]. Prior to statehood in 1959, the Alaska Native Claims Settlement Act (ANCSA) in 1971, and other influential factors (e.g., globalization [26,27]), many Alaska Natives followed a nomadic or semi-nomadic way of life to hunt and gather food that produced minimal waste. Waste composition changed with the development of permanent villages and increased consumption of imported goods, necessitating, a waste management system to protect human health and the environment of their communities. However, communities have limited access to funding for managing existing solid waste infrastructure or for constructing new infrastructure. Many Alaska Native communities obtain funding through grants from the U.S. Environmental Protection Agency’s (EPA’s) Indian General Assistance Program (IGAP), which supports solid waste operations. However, IGAP is intended for Tribes to develop and administer a multitude of environmental capacities, including legal, technical, administrative, financial management, information management, environmental needs assessment, and public education programs. These limited funding options constrain Alaska Native communities’ solid waste management abilities, leading to concerning consequences for environmental and human health.

Solid waste issues are also exacerbated by recent Arctic weather trends that destabilize the region’s ecosystems, creating additional challenges for landfill management. Specifically, increasing air temperatures have caused reductions in sea ice and snow cover and greater thawing of glaciers and permafrost, which have together produced a vicious cycle of elevated solar energy absorption and atmospheric warming [28,29,30]. These changes can contribute to greater landfill contaminant exposure and waste leachate mobility, since many landfills have historically relied on “Freezeback” systems, where permafrost and cold temperatures keep buried waste frozen year-round, or have used permafrost as a barrier between waste and the surrounding environment. However, permafrost temperature has risen between 0.28° and 0.47 °C per decade since 2000 on the North Slope of Alaska. Furthermore, the active layer thickness (the top layer of soil that thaws during the summer and freezes again in winter) increased by 11% from 1995 to 2013 [31]. As a result, thawing permafrost can form taliks, which are zones of year-round unfrozen ground within, or above permafrost caused by thermal and hydrologic factors. This leads to greater ground saturation and interaction between surface and groundwater. Taliks not only increase maintenance needs for fencing around landfills on uneven and moving ground but may also create contaminant pathways through animal scavenging or water and soil contaminant transport from Class III landfills that are unlined and lack leachate collection systems.

In this way, shifting waste composition and climate-driven ground and hydrologic changes may have synergistic effects, contributing to environmental and human health risks in rural Alaska through greater waste leachate mobility. Consequently, greater knowledge of landfill contaminant risks in rural Alaska is necessary for infrastructure planning, response, and remediation. We conducted a scoping literature review to gather the existing information from peer-reviewed literature and gray literature in public databases on the risks of landfill contaminants in rural Alaska. This review had three major aims: (1) Develop a conceptual model of how Arctic municipal landfills may generate environmental and human health impacts; (2) Quantify the available information on environmental impacts of Class III landfills in rural Alaska communities; and (3) Assess the human health impacts of Class III landfills in rural Alaska. Rural Alaska communities experience disproportionate challenges in solid waste management and will encounter the greatest, most urgent consequences from increasing temperatures due to the design and operation of Class III landfills. For Alaska Natives, these challenges may affect nearby subsistence activities in ways that increase food insecurity and cumulative stress, endangering community health. For these reasons, a better understanding of the environmental and human health risks posed by solid waste contaminants can be used to reduce the undue burden on Alaska Native communities and proactively protect both environmental and human health.

## 2. Materials and Methods

We conducted a scoping literature review using the Sciome Workbench for Interactive-computer-Facilitated Text-mining (SWIFT)-Active Screener to find relevant literature from the search results. We also used EPA’s Health and Environmental Research Online (HERO) database to document literature search results. In October 2021, we worked with HERO librarians to develop a set of search terms and strategies to investigate our study aims. We identified potentially relevant, peer-reviewed publications by searching for these terms in three literature databases (PubMed, Web of Science, & JSTOR) and several public databases for gray literature, including the National Service Center for Environmental Publications (NSCEP), the National Institute of Environmental Health Sciences (NIEHS), the Alaska Department of Environmental Conservation (ADEC), the United States Geological Survey (USGS), Research Gate, the Agency for Toxic Substances and Disease Registry (ATSDR)—Public Health Assessments & Health Consultations—Alaska, ProQuest—Agricultural & Environmental Science Collection, and the National Indian Law Library (see Appendix A for search terms by databases).

These searches yielded 3353 unique results. Given the large number of references, we used SWIFT-Active Screener to reduce the amount of time required to screen references at the title/abstract level. SWIFT-Active Screener is a collaborative, web-based systematic review software that uses machine-learning algorithms to automatically prioritize sources as they are reviewed, using user feedback to push the most relevant sources to the top of the list [32]. The “Active Learning” model updates in real time as users include or exclude each source, while a second model estimates the number of relevant sources remaining from the list of unscreened documents. These models work in tandem to identify the majority of relevant references after the user has screened only a subset of the total documents [32]. In the SWIFT-Active Screener, we provided 18 “positive seed” references prior to screening process to inform the machine-learning algorithm. The positive seeds were references that the authors had previously read and knew would fall within the scope of this literature review. In November 2021, authors AMN and KS both test screened 20 of the 3353 references to develop consistent screening methods.

**Figure 2:**
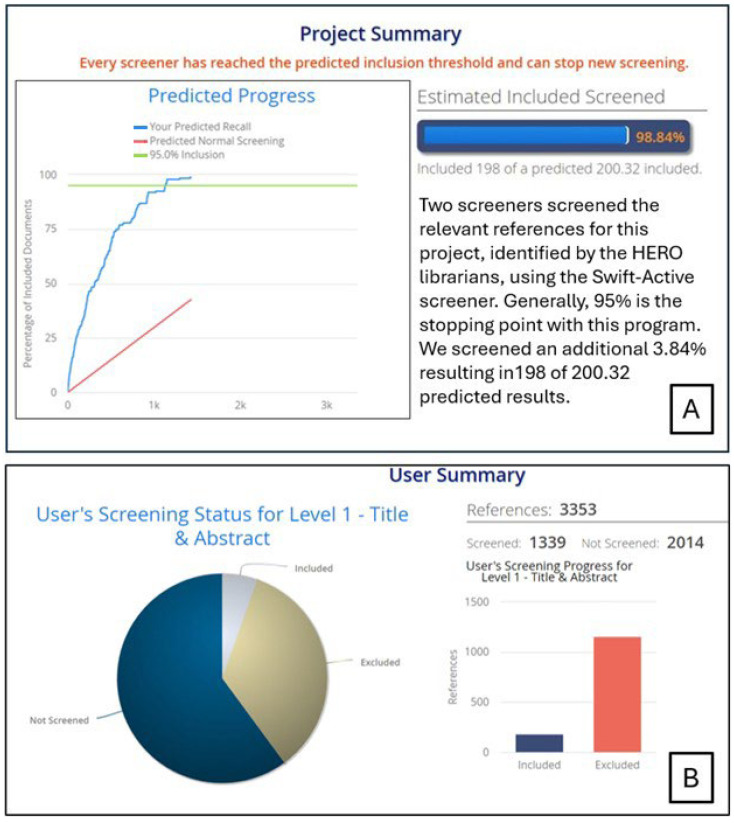
SWIFT-Active Screener results for the project. (**A**) Project Summary presents the predicted progress of screeners AMN & PJ in identifying relevant references. The red line shows the predicted normal screening, without using the machine learning algorithm to inform screening order; the blue line shows the predicted recall with the machine learning algorithm; the green line shows the 95% threshold for screening. (**B**) User Summary shows the total number of references available in the HERO database and the number that were screened and not screened in SWIFT-Active Screener.

After the test screening, authors AMN and PJ screened the 3353 references based on titles and abstracts starting on 2 December 2021. On 4 January 2022, we reached the recommended 95% inclusion threshold (i.e., the point at which 95% of the projected number of relevant references had been included) with only 42% of the references screened. We screened an additional 8 references for a final inclusion threshold of 98.84% (198 included out of a predicted 200.32) (see Figure 2).

**Figure 3:**
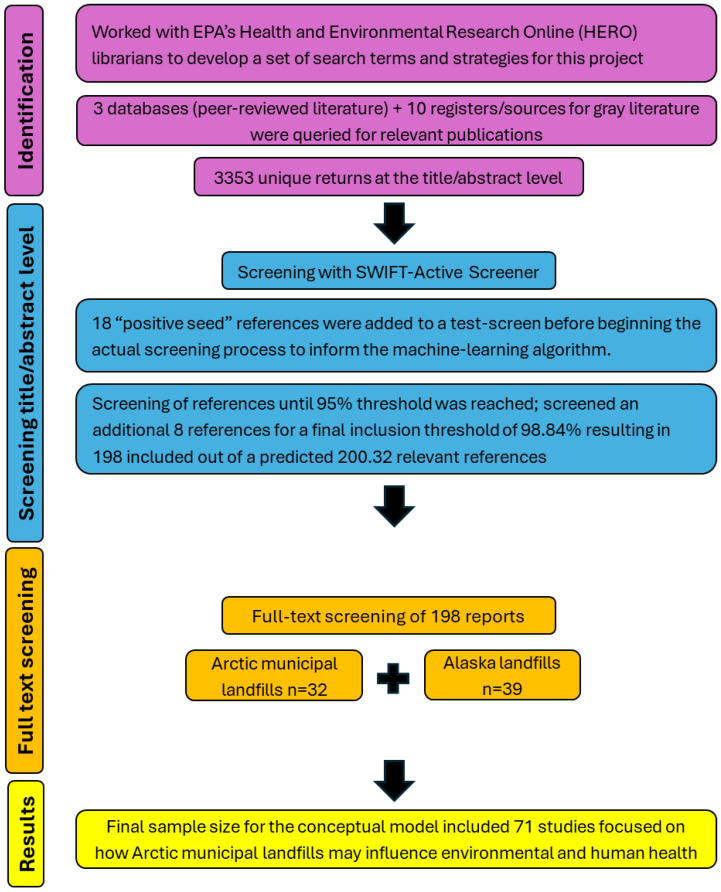
Diagram of the review process.

Author CC completed the full-text screening on the 198 references included after title/abstract screening. Detailed inclusion/exclusion criteria are described in Appendix B. Briefly, the reference had to be published in English and report data on human or environmental health related to solid waste issues in the Arctic to be included in the conceptual model. For the literature review portion of the analysis, we applied the same criteria with the additional restriction that data must have been collected in Alaska. Figure 3 illustrates the flow of information across each review stage. From the Alaska-specific references, we extracted information on population, location, sample size, waste disposal type, climate/transport processes, inclusion of Indigenous knowledge, and summaries of key points (Table 1 and Table 2). This scoping review was reported according to the Preferred Reporting Items for Systematic Reviews and Meta-Analyses extension for Scoping Reviews (PRISMA-ScR; see Supplementary Material) [33]. It was not pre-registered.

## 3. Results

The final sample size for the conceptual model included 71 studies, spanning 46 years (1975–2021), that investigated how Arctic municipal landfills may influence environmental and human health (Aim 1). The total number of references focused on Alaska landfills encompassed 39 studies, with 33 references addressing environmental health (Aim 2) and six references addressing human health (Aim 3).

### 3.1. Conceptual Model

**Figure 4:**
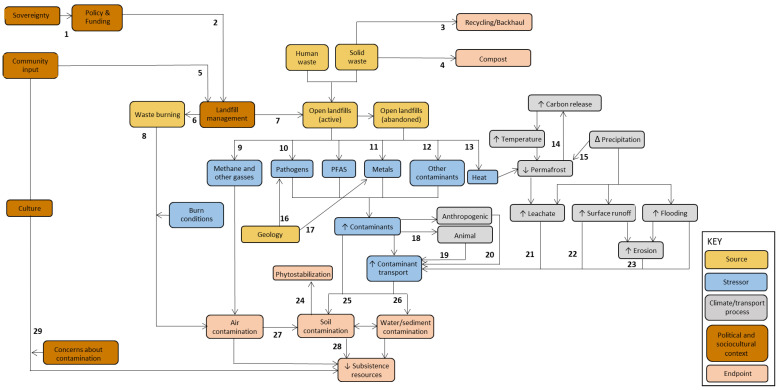
Conceptual model of the potential pathways that landfill contaminants may affect human health and the environment in the Arctic region. The citations for each numbered pathway are as follows: 1 [34,35]; 2 [24,25,36,37,38,39]; 3 [40]; 4 [41]; 5 [42,43,44,45]; 6 [23,46,47,48,49,50,51,52]; 7 [23,48,53,54,55,56,57,58]; 8 [59,60], 9 [61]; 10 [18,62]; 11 [18]; 12 [18,62]; 13 [61,63,64,65]; 14 [66,67,68,69,70]; 15 [71]; 16 [72]; 17 [72]; 18 [73,74,75]; 19 [74,76]; 20 [77,78]; 21 [79,80,81,82,83,84,85]; 22 [77,86]; 23 [87]; 24 [88]; 25 [89]; 26 [90,91,92]; 27 [93]; 28 [94]; 29 [23,95].

The conceptual model visualizes the potential pathways by which landfill contaminants may affect human health and the environment in the Arctic region (Figure 4). Relationships supported by published literature are bolded and numbered, with the references for each number documented below the model. The relationships investigated in the 71 references were not evenly distributed across processes in the conceptual model. Five or more references investigated the effects of landfill management, waste burning, heat generation from solid waste, carbon release from permafrost, leachate characterization, and water/sediment contamination. However, we found few references investigating other pathways in the model, such as those related to erosion, soil and water contamination, flooding, and Alaska Native culture, food systems, and subsistence resources. We also found few references that investigated alternative ways to prevent the accumulation of waste in landfills, such as reuse, composting, or backhaul.

### 3.2. Environmental Results

The 33 studies that investigated the environmental effects of landfill contaminants in Alaska centered on six topics: (1) Water, (2) Soil, (3) Air, (4) Heat, (5) Microbes, and (6) Animal garbage consumption. These studies are summarized in Table 1 and the sections below. All studies are from Class III landfills unless otherwise noted.

**Table 1:** Summary information of the studies investigating environmental health.

Author (Year)	Environmental Impact	Source Type	Main Outcome	Location	Sample Size	Main Finding
Ahlstrom et al. (2018) [73]	Microbes	Scientific Article	*E. coli*	Soldotna landfill in southcentral Alaska	27 *E. coli* isolates (13 from 20 bald eagles, 14 from 56 gulls)	Some *E. coli* isolates had sequence types associated with human infections and contained clinically relevant resistance genes. Gulls and eagles demonstrated some genetically unrelated isolates with identical resistance profiles, while other isolates had identical core genomes and different resistance profiles. These results suggest bacterial strain sharing between species as well as horizontal gene transfer, with landfills serving as a source for antimicrobial resistance (AMR) acquisition or maintenance.
Ahlstrom et al. (2019) [74]	Microbes	Scientific Article	*E. coli*	Kenai Peninsula	17 gulls, 139 Pacific salmon	AMR *E. coli* prevalence varied across time and space among gulls tracked longitudinally, although the landfill and the lower Kenai River had the highest prevalence. They found no evidence of *E. coli* in salmon from personal-use dipnet fisheries. These results suggest that gulls acquire AMR *E. coli* at anthropogenically disturbed sites and then transport it as they migrate.
ATSDR (2019) [91,96]	Water	Report	Heavy metals	Port Heiden, Alaska	Varied depending on part of study	Based on the physical and chemical hazards in Port Heiden, the authors found that people could be injured by surface debris. They also found that school drinking water met standards after treatment and called for greater information to assess residential wells, contamination from landfills/military, vapor intrusion, and contamination of subsistence foods.
ATSDR (2014) [94]	Soil	Report	PCBs	Port Heiden, Alaska	N/A	The low PCB levels detected in the relay station soil, roadway, and small animals were not expected to cause health effects. Crowberries had low but safe levels of PCBs; however, they recommended that crowberry consumption should be avoided. The area with foundation cover soils and Pad Grid 1—which is infrequently visited by residents—had high PCB levels. They could not assess risk from marine sources.
Barnes (2011) [62]	Water, microbes	Report	Environmental contaminants, *E.coli*, and *Enterococci*	Rural Alaska	4 communities	*E.coli* and *Enterococci* were present in all waste-impacted surface and subsurface water samples. They also detected seven pharmaceutical compounds in the sewage lagoon and landfill-impacted water.
Brunett (1990) [79]	Water	Report	Physiochemical parameters, environmental contaminants	Merrill Field Landfill, Anchorage, Alaska	444 measurement stations and 20 wells	Leachate from the closed Merrill Field landfill did not appear to be contaminating the creek that flows through the area. However, leachate was transported to the southern wetlands through groundwater. Contaminants reached wetlands as far as 2200 feet away based on aquifer and well sampling, although levels were below EPA drinking water standards.
Chambers (2005) [77]	Microbes	Thesis	*E. coli*, total coliform, *Giardia lamblia*, and *Cryptosporidium parvum*	Rural Alaska	Varied depending on part of study	Surface water flow transported bacteria to the community during spring thaw, but flow from the landfill did not contribute to contamination in town. Inside homes, fecal bacteria were found on water dippers, kitchen counters and floors, and in washbasin water. *Giardia* was found at the landfill.
Chambers et al. (2009) [78]	Microbes	Scientific Article	*E. coli* and total coliform	Rural Alaska	19 boot samples, 12 road/off road sample pairs, 4 puddle samples, 45 ATV samples, 10 tarp samples	Shoes transported fecal contaminants inside homes. Fecal contamination was also observed in puddles on the roads and on ATV tires, which suggests vehicle use is one transport route for fecal contaminants.
Downey (1990) [82]	Water	Report	Physiochemical parameters, heavy metals	Fairbanks, AK	22 wells, 2 rivers	Leachate was flowing to the Northwest of the Fairbanks-North Star Borough landfill. It remained near the water table. Chemical data showed elevated levels of several ions in the leachate plume, but they fell to background within a short distance from the landfill, suggesting water-supply wells would not be affected.
Flynn (1985) [83]	Water	Report	Physiochemical parameters, heavy metals	Fairbanks, AK	22 wells, 2 rivers	Several wells in the Fairbanks Sanitary Landfill showed high concentrations of chloride, iron, and manganese compared to background levels. They also had relatively low pH and dissolved-oxygen concentrations. However, these constituents and properties fell to background levels in wells north and west of the landfill.
Gilbreath (2004) [97]	Air	Thesis	Adverse birth outcomes, self-report health symptoms	Rural Alaska	1225 individuals (self-report), 10,073 individuals (birth outcomes), 10,360 individuals (congenital anomalies)	Self-reported health symptoms were associated with odor complaints, burning trash, number of visits to the landfill, subsistence practices, and residing within a 1/2 mile of a dumpsite. Women living in intermediate and high hazard dumpsite communities had lower birthweight, shorter gestation length, and greater congenital defects.
Glass (1986) [84]	Water	Report	Physiochemical parameters	Connors Bog Area, Anchorage, AK	36 wells	Leachate was found beneath and near the abandoned landfill, and it contained elevated levels of dissolved solids, dissolved chloride, and total organics. The leachate was limited to < 500 feet from the landfill’s edge, and they found no evidence of leachate in the lake.
Hanson et al. (2008) [63]	Heat	Scientific Article	Temperature	Alaska, Michigan, British Columbia, New Mexico	4 landfill sites with 700 sensors (measured weekly)	They measured landfill temperatures at different depths in four landfills. The warmest section was the central part of the middle third fraction. Higher areas were more similar to air temperature. The highest temperatures were observed in Michigan, followed by British Columbia, New Mexico, and Alaska.
Hanson et al. (2010) [98]	Heat	Scientific Article	Temperature	Alaska, Michigan, British Columbia, New Mexico	4 landfill sites with 700 sensors (measured weekly)	They measured landfill temperatures at different depths in four landfills. The warmest part was the central part of the middle third fraction. Higher areas were more similar to air temperature. Temperatures were greatest in Michigan, then British Columbia, New Mexico, and Alaska. Anaerobic decomposition was associated with greater temperatures, temperature increases, and heat gain. Insulating materials applied over covers decreased temperature variation.
Liu (2007) [61]	Heat	Thesis	Temperature	Michigan, New Mexico, Alaska, and British Columbia	4 landfill sites that included 609 temperature sensors (measured weekly) and 327 gas sensors (measured monthly)	Temperatures and methane at shallow depths or near landfill edges fluctuated seasonally. Both measures were stable in the middle and lower portions near the base. Their temperature and gas-release models accurately represented field conditions.
Mutter (2014) [18]	Water, microbes	Thesis	Environmental contaminants, *E.coli*, and *Enterococci*	Rural Alaska	5 communities (5 dumps, 2 sewage systems)	*E.coli* and *Enterococcus* sp. were present in waste-impacted water and soil samples. They also observed heavy metal migration into nearby freshwater sources and found pharmaceuticals, phthalates, and benzotriazole in waste-impacted water samples.
Mutter et al. (2017) [86]	Microbes	Scientific Article	*E.coli* and *Enterococci*	Ekwok, White Mountain, Fort Yukon, Allakaket	4 communities	Although all samples indicated high site-specific variability, both *E. coli* and *Enterococcus* sp. preferentially attached to and migrated with soil particles in surface waters. Additionally, both were transported off-site in snowmelt runoff. *Enterococcus* sp. had greater viability in cold conditions.
Naidu (2003) [89]	Soil	Scientific Article	Heavy metals	North Slope, Alaska	2 sites	In the urban site, V increased each decade and Ba from 1986 to 1997. In both sites, levels of all metals were similar to unpolluted marine environments. Less than 1% of Hg was methylated, and percentages of elements bound in the non-lithogenous phase varied (50% Mn, 25–35% Co, 15–20% Zn, Cu, Ni, 10% V & Fe, and <3% of Cr).
Nelson (1984) [80]	Water	Conference Paper	Water Quality	Anchorage, Alaska	N/A	They calculated that incipient entry of pollutants into the aquifer would begin 80 years after leachate began migrating downward and would only reach “full strength” of breakthrough after 250 years.
Patterson et al. (2012) [85]	Water, microbes	Report	Physiochemical parameters, heavy metals, environmental contaminants, *E. coli*, and *Enterococcus*	Rural Alaska	5 villages	They did not find evidence that landfill leachate was contaminating drinking water. However, they observed that microbial pathogens and aluminum levels were elevated in the leachate and should be monitored in treated drinking water and source waters.
Peirce & Van Daele (2006) [75]	Animal garbage consumption	Scientific Article	Behavioral observations	Dillingham, Alaska	70 brown bears	Seventeen bears were predictable users of the Dillingham Landfill and had temporal patterns of use. Between four and 33 bears visited each night, with peak use occurring in July. Subadult activity peaked in June, male activity in June and August, and females with cubs in September. The most socially dominant bears fed the most from the landfill.
Solid Waste Program, Alaska DEC (2015) [87]	Soil	Report	Erosion risk	North & West AK coasts & ≤ 300 miles upriver, Aleutian Islands	716 sites in 124 communities	Based on erosion risk and contaminant risk scores, they wrote Detailed Action Plans for the sites with the highest risk of eroding and distributing contaminants.
Weiser & Powell (2010) [76]	Animal garbage consumption	Scientific Article	Garbage in diet samples	Barrow, AK	Nonbreeding colony: 193 samples in 2007 and 248 2008. Breeding colony: 46 samples in 2007 and 403 in 2008.	Breeding adult gulls ate less garbage than nonbreeding gulls. Breeding gull samples showed no garbage change 2007–2008 while nonbreeding gulls consumed less garbage in 2008 than in 2007. Overall, garbage remained a large part of the diet in 2008.
Yesiller et al. (2005) [65]	Heat	Scientific Article	Temperature	Alaska, Michigan, British Columbia, New Mexico	4 landfill sites with 355 temperature sensors (measured weekly) & 238 gas sensors (measured monthly)	Temperatures at shallow depths and near the edges of the landfills were similar to seasonal temperature variations. Deep and central locations had elevated temperatures compared to air and ground temperatures. Waste temperatures also decreased near the base. Peak Heat Content values were 12.5–47.8 °C/day. The highest values for temperatures, gradients, heat content, and heat generation were Michigan, followed by British Columbia, Alaska, and New Mexico.
Yesiller et al. (2008) [64]	Heat	Scientific Article	Temperature	Alaska, Michigan, British Columbia, New Mexico	4 landfill sites	Landfill cover temperature varied seasonally with air temperature; it also demonstrated amplitude decrement and phase lag with depth. They found that warmer waste underneath landfill cover was associated with warmer cover and less frost penetration. Maximum and minimum temperature ranges were 18–30 °C and 13–21 °C. Average temperature was 13–18 °C at 1 m and 14–23 °C at 2-m depths. They found that frost depths were approximately 50% of those for soils at ambient conditions. Heat mainly flowed upward in the covers. Cover gradients varied between 18 & 14 °C/m.
Zenone (1975) [81]	Water	Scientific Article	Physiochemical parameters, heavy metals	Alaska, Michigan, British Columbia, New Mexico	18 wells across 3 landfill sites	Leachate was detected in the ground water near two sites. At these sites, the water table was near land surface and waste was deposited at or below the water table. The leachate plume seemed to attenuate within the landfill or close by at the first site. They did not find leachate at a third site where waste disposal occurs above the water table.

#### 3.2.1. Water

Eight studies investigated water quality issues related to solid waste in unlined landfills without leachate collection systems. These studies focused on physiochemical parameters, heavy metals, or other contaminants. While some of the included studies investigated leachate in Class I landfills [73,74,76,77], these landfills lacked leachate collection systems at the time, so their results are relevant for understanding potential environmental contamination from present-day Class III landfills.

Six of the studies investigated physiochemical water quality indicators (e.g., dissolved solids, total organics, dissolved organic carbon, alkalinity, dissolved oxygen, dissolved organic nitrogen, nitrite, nitrate, dissolved inorganic nitrogen, sulfate, phosphate, chloride, and fluoride) across six distinct landfills (see Table 1 for study sites). Of these water quality indicators, researchers found elevated levels within landfills or leachate plumes for dissolved solids [81,82,84], chloride [82,83,84,85], silica [82], alkalinity [82], organic carbon [81], dissolved oxygen [83], lower pH [83], total organics [84], and sulfate [85]. Levels of these contaminants returned to background levels within short distances [82,83,84], and specifically within 500 feet of the landfill’s edge in Glass (1986) [84]. In Brunett (1990), leachate moved deeper between the two study years (1985 and 1986); however, despite this movement, leachate remained concentrated in the top 50 feet below the landfill [79]. Additionally, two of the three landfill sites studied in Zenone (1975) were located in sites where the water table was near the land surface and where waste was deposited at or below the water table [81]. Levels of organic carbon and dissolved solids were elevated only in these two sites, suggesting that saturated waste was contributing to leachate production.

Six studies also examined heavy metal concentrations in leachate, including aluminum, arsenic, beryllium, cadmium, chromium, iron, lead, manganese, uranium, zinc, vanadium, barium, mercury, and nickel [81,82,83,85,91,96]. Researchers reported elevated levels within leachate or leachate plumes for manganese [81,82,83], iron [81,82,83], barium [82], arsenic [82], and aluminum [85]. Similar to traditional measures of water quality, metal concentrations decreased to background levels within a short distance of the landfill [81,83]. One report compiled longitudinal water quality data to assess contaminant risk to residents of Port Heiden, Alaska—a city containing landfills and land formerly used by the U.S. military [91,96]. They determined that school drinking water met primary drinking water regulations after treatment, although copper and arsenic levels increased rapidly. Therefore, they recommended that the filtration system must be sufficiently maintained to prevent copper and arsenic exceedances. Additionally, diesel range organics were detected in some wells, but there was insufficient information to assess risk.

Three studies investigated other contaminants, including benzene, trichloroethylene, vinyl chloride, other volatile and semi-volatile organic compounds (VOCs and SVOCs), phthalates, benzotriazole, and pharmaceuticals [18,79,85]. Brunett (1990) found elevated levels of benzene, trichloroethylene, and vinyl chloride in leachate samples [79]. Mutter (2014) investigated landfills in five communities, and found detectable levels of pharmaceuticals, benzotriazole, and phthalates in waste-impacted water samples (leachate) [18]. All VOCs and SVOCs were undetectable in Patterson et al. (2012) [85].

Taken together, these studies suggest that Class III landfills release a variety of contaminants that can leach into surface and groundwater, including heavy metals (manganese, iron, barium, arsenic, and aluminum) [18,81,82,83,85]. In most studies, contaminant concentrations returned to background levels near the landfills. However, not all studies assessed the geographic spread of contaminants, and more work is needed to assess contaminants such as benzene, trichloroethylene, vinyl chloride, pharmaceuticals, benzotriazole, and phthalates at other landfill sites to determine whether they are consistently elevated in Class III landfills.

#### 3.2.2. Soil

Five studies explored how landfill contaminants may influence soil quality. Mutter (2014) and Mutter et al. (2017) report on fecal bacteria (e.g., *Escherichia coli)* in soil; these studies are described in the “Microbes” subsection below [18,86]. The remaining three studies focused on heavy metals, polychlorinated biphenyls (PCBs), and erosion risk.

One study compared heavy metal contamination in two lagoons, one near Barrow, Alaska (an “urbanized” site), and another near Arctic National Wildlife Refuge (a “pristine” site), for 30 years [89]. They found that vanadium levels increased each decade in the urban site, and barium increased from 1986 to 1997. Levels of all metals across both sites were comparable to those in unpolluted marine environments. However, some contaminants, including vanadium, chromium, nickel, and total mercury at the “urbanized” site and vanadium and manganese at the “pristine” site, were greater than measurements from the nearshore area of a petroleum-related industrial area. While this project did not examine landfill contaminants directly, the authors speculated that sources of these metals in the environment may have included municipal solid waste. Additionally, as the landfill did not drain to the lagoon, further research would be needed to clarify the transport route to support their interpretation of landfill contaminant impacts on soil.

One report investigated PCBs in soil and subsistence foods in areas and landfills formerly used by the U.S. military in Port Heiden, Alaska [94]. Researchers found very low levels of PCBs in soil around the Radio Relay Station, the roadway, and in small grazing mammals, ducks, and sea birds. The area containing foundation cover soil and Pad Grid 1 had high levels of PCBs, but residents were not at risk since they did not visit these sites. They also found that crowberries had detectable, low levels of PCBs. Due to the previous military activity and radio relay station, the measured PCB exposure is likely not generalizable to other Class III landfills.

Another report investigated erosion risk in rural Alaska communities for sites of environmental concern, including landfills [87]. This study did not conduct environmental sampling but did calculate contaminant risk based on site and environmental characteristics. They assessed and ranked sites within communities based on erosion types and symptoms, probable contaminants, and potential for human and environmental exposure, identifying 20 sites that ranked in the top 25% for both erosion risk and contaminant risk.

#### 3.2.3. Air

One study investigated the effects of solid waste on air quality [97]; this study is discussed below under Section 3.3.2. No studies on air quality composition from waste decomposition processes or waste burning practices were found.

#### 3.2.4. Heat

Five studies investigated landfill temperature and heat generation from solid waste decomposition in Class I landfills [61,63,64,65,98]. These studies used data from the same four landfills in Alaska, Michigan, Mexico, and British Columbia [61,63,64,65,98]. They consistently found that methane levels and waste temperature varied seasonally—similar to variations in air temperature—for waste at shallow depths or close to the edges of the landfill. Waste in the central area of the landfill and in the vertical middle third was the hottest, and temperatures in this core were relatively stable. Temperatures decreased again near the base, or deepest part, of the landfills. Environmental conditions and precipitation at the time of waste placement also influenced landfill temperatures: waste placed on hotter days or when the landfill was wet from rainfall remained at greater temperatures than waste placed on cold or dry days [61,63,65,98]. However, these temperature dynamics from Class I landfills are likely not generalizable to Class III landfills in rural Alaska communities.

#### 3.2.5. Microbes

Eight studies investigated solid waste as a source of microbe exposure in Class I and Class III landfills. Five studies compared samples from locations near Class III waste sites and control areas, observing detectable levels of *E. coli* and *Enterococcus* species in all waste-impacted surface water, subsurface, and soil samples [18,62,77,85,86]. Of these sources, Barnes (2011) and Mutter (2014) report the same data for fecal bacteria in surface and subsurface waters in four communities [18,62]; Patterson et al.’s work (2012) was a parallel project involving the same four communities plus a fifth community [85]. Additionally, Mutter (2014) and Mutter (2017) report the same data about soil fecal bacteria [18,86]. Fecal bacteria levels exceeded EPA standards for the samples collected near dumpsites in four of these communities [18,62,85,86]. Generally, levels of *E. coli* and *Enterococcus* species were undetectable 50 m or further downgradient from the waste sites [18,62,85,86], although fecal bacteria could be transported offsite (>50 m away from the waste site) when snowmelt occurred in the spring [18,86]. Mutter et al. (2017) used a lysimeter and freezer experiments to investigate differences in fecal bacteria snowmelt transport processes and cold climate survivability. These experiments, revealed that *Enterococcus* species had greater and more sustained viability in cold conditions, while *E. coli* was less persistent [86]. Chambers (2005) also investigated two pathogens (*Giardia lamblia* and *Cryptosporidium parvum*) in a subset of their surface water and fecal samples (dog and human) [77]. They found *Giardia lamblia* in a surface water sample from the community dumpsite, but not in human or dog fecal samples.

Two studies investigated fecal bacteria transport routes that could contribute to human exposure in communities with Class III landfills and honeybucket use [77,78]. Chambers (2005) investigated indoor home contamination in one rural Alaska community that was unplumbed, necessitating haulage of water and sewage [77]. The boardwalk into town was also used for honeybucket lagoon disposal. They detected fecal bacteria on water dippers, kitchen counters, floors, and in washbasin water. They also found that tires and shoes tracked fecal contamination from the dump, although tires did not do so consistently. Chambers et al. (2009) conducted experimental trials to further investigate the efficiency of boots, tires, and other surfaces as transport materials or routes for fecal bacteria [78]. Boots consistently transported fecal contamination inside homes. They also found fecal contamination in puddles and on ATV tires, which supports vehicle use as a pathway for fecal contamination to reach community homes. Compared to Chambers (2005), tires were much more effective in transporting fecal bacteria, which may have occurred because they conducted the experiment in a wetter season than the original study [77]. Additionally, they found no evidence that tires consistently transferred fecal bacteria to the boardwalk. Together, these results suggest that fecal bacteria transfer most efficiently to soft, moist surfaces [78].

The final two studies examined how animals may contribute to microbial transport in the environment based on data from Class I landfills [73,74]. These studies used gull and bald eagle feces to investigate the prevalence and sources of antimicrobial resistant (AMR) *E. coli.* In both studies, AMR *E. coli* was identified in fecal samples. Ahlstrom et al. (2018) collected samples at only one landfill, while Ahlstrom et al. (2019) tracked gulls longitudinally across four sites [73,74]. In Ahlstrom et al. (2019), the landfill study site and the lower Kenai River site had the highest prevalence of AMR *E. coli*, although prevalence varied across sites and over time [74]. Together, these studies suggest that birds such as eagles and gulls acquire AMR *E. coli* at landfills by consuming waste and can then spread it to other locations via excretion. Since the AMR *E. coli* strains studied in these projects are sequence types associated with human infections and clinically relevant resistance genes, the role of birds and other animals in spreading AMR *E. coli* could have public health implications. Additionally, Ahlstrom et al. (2018) observed that some gulls and eagles had nearly genetically identical *E. coli* isolates, which may indicate either that both species acquire *E. coli* from the same source or that inter-species transmission is occurring [73].

#### 3.2.6. Animal Garbage Consumption Patterns

Two studies explored solid waste effects on the environment through animal consumption of garbage [75,76]. Peirce & Van Daele (2006) conducted behavioral observations on 70 brown bears (*Ursus arctos*) that visited the Dillingham Class II landfill from May to September 1997 [75]. They found temporal patterns in bear foraging in the landfill waste, with peak use occurring in July when their natural foods are in low availability. Weiser & Powell (2011) studied 890 diet samples from two colonies of glaucous gulls in Barrow—one breeding and one non-breeding [76]. The samples were collected during two time periods: 2007, when waste was disposed of in a landfill, and 2008, when waste was incinerated. Breeding gulls ate more garbage than nonbreeding gulls. Breeding gull samples showed no significant change in garbage consumption between 2007 and 2008, while nonbreeding gulls ate less garbage in 2008 than in 2007. For both colonies, garbage continued to constitute a large proportion of gulls’ diet in 2008; waste incineration alone did not meaningfully decrease gull garbage consumption. Neither study investigated contaminant concentrations. More research is needed to elucidate the exposure and transport dynamics associated with animal garbage consumption, particularly because inter-species transmission of clinically relevant microbes could have important public health implications.

### 3.3. Human Health Results

The six studies that investigated relationships between human health and landfill contaminants focused on either infant health (two studies) or population health (four studies) (see Table 2). No studies investigated landfill contaminants and child health, although Central Council of Tlingit and Haida Tribes and Zender Group (2003) discussed the potential indirect exposure of children using the landfill or dump as a playground, which occurred in 14% of surveyed Alaska Native communities [23].

**Table 2:** Summary information of the studies investigating human health.

Author	Study Focus: Adult or Child Health	Article Type	Main Outcome	Location	Sample Size	Summary
Central Council of Tlingit and Haida Tribes & Zender Environmental (2003) [23]	Adult	Report	Self-report health symptoms	Rural Alaska	101 Alaska Native communities and 1225 individuals	Most waste disposal sites in Alaska Native Villages were open dumps with little management. Due to limited solid waste management services, most residents dumped their own waste. Improper disposal of waste, such as unsorted burning and uncovered antifreeze, also contributed to the health risks.
Gilbreath (2004) [97]	Adult & Child	Thesis	Adverse birth outcomes, self-report health symptoms	Rural Alaska	1225 individuals (self-report), 10,073 individuals (birth outcomes), 10,360 individuals (congenital anomalies)	Worse self-reported health symptoms were associated with residing within a 1/2 mile of a dumpsite, odor complaints, burning trash, number of visits to the landfill, and subsistence practices. Women living in intermediate and high hazard dumpsite communities had lower birthweight, shorter gestation length, and greater congenital defects.
Gilbreath & Kass (2006) [99]	Child	Scientific Article	Adverse birth outcomes	Rural Alaska	10,073 individuals	Villages with intermediate and high hazard dumpsites had significantly greater percentage of infants with low birth weight and intrauterine growth retardation. Specifically, infants weighed 55.4 g and 36 g less when their mother was in the high exposure group compared to the low and intermediate exposure groups, and this effect was even larger when limited to Alaska Native mothers only.
Gilbreath & Kass (2006) [100]	Child	Scientific Article	Adverse birth outcomes	Rural Alaska	10,360 individuals	Villages with intermediate and high hazard dumpsites did not have statistically significantly greater fetal and neonatal death or congenital anomalies. Mothers living in villages with high hazard dumpsites were four times more likely to have congenital anomalies classified as “other”.
McBeth (2010) [101]	Adult	Thesis	Respiratory infection deaths	Rural Alaska	196 villages	High household size and low household income predicted greater pneumonia/influenza deaths in Alaska Native Villages. Tuberculosis deaths were associated with residence in certain areas and the type of heating fuel used in the home. Lastly, infectious disease deaths were positively associated with a high percentage of Alaska Natives in the population, large household, low percentage below poverty, and lack of healthcare within the village.
Zender et al. (2003) [24]	Adult	Report	Self-report health symptoms	Rural Alaska	101 Alaska Native communities and 1225 individuals	Most waste disposal sites in Alaska Native Villages were open dumps with little management. Due to limited solid waste management services, most people dumped their own waste. Improper disposal of waste, such as unsorted burning and uncovered antifreeze, also contributed to the health risks.

#### 3.3.1. Infant Health

Only two studies empirically investigated the effects of solid waste management on infant health [99,100]. Gilbreath and Kass (2006) used birth records (n = 10,360) from 197 Alaska Native communities containing dumpsites that were potentially hazardous to human health, based on Alaska Native Tribal Health Consortium (ANTHC) scoring [100]. They found that communities with intermediate and high hazard dumpsites did not demonstrate significantly greater fetal death, neonatal death, or congenital anomalies. Scores were based on various risk factors, including landfill waste contents, average rainfall, distance to drinking water aquifer and domestic water source, site drainage, potential to create leachate at the site, accessibility and exposure to the public and vectors, frequency of burning, and degree of public concern over the site [102]. However, congenital anomalies trended higher in communities with more hazardous dumpsites. When considering specific congenital anomalies, they observed that infants in communities with high hazard dumpsites were more than four times more likely to have congenital anomalies classified as “other” compared to communities with low or intermediate hazard dumpsites [100].

Gilbreath & Kass (2006) used the same methods to investigate the prevalence of low birthweight and intrauterine growth restriction (n = 10,073 birth records) [99]. They found that communities with intermediate and high hazard dumpsites had a significantly greater percentage of infants with low birth weight and intrauterine growth restriction than infants in low hazard dumpsite communities. Specifically, infants weighed 36 g less in the high exposure group and 55.4 g less in the intermediate group when compared to the low exposure group, and this effect size was even greater when the results were limited to infants of Alaska Native mothers only. Unexpectedly, infants from communities with intermediate hazard sites had a greater risk than high hazard sites for both lower birthweight and intrauterine growth restriction; this result requires more investigation but may be due to uncontrolled covariates or the limited precision of the exposure measurements.

#### 3.3.2. Population Health

Two studies and two reports investigated solid waste management and population health in Alaska [23,24,97,101]. McBeth (2010) used data from the U.S. Census, the Alaska Alcohol Beverage Control Board, and the Alaska Native Tribal Health Consortium (ANTHC) to investigate predictors of infectious disease in Alaska Native communities [101]. Among other predictors, they included census data on the percentage of homes with complete plumbing or kitchens, since a lack of this infrastructure leads to honeybucket use and potentially greater microbial exposure (see Microbes subsection above), particularly as solid waste and human waste are comingled in at least 30% of rural communities [23,24]. However, analysis showed that complete plumbing and/or kitchens were not a significant predictor of pneumonia/influenza deaths, tuberculosis deaths, or overall infectious disease deaths. Gilbreath (2004) found that traditional diets were associated with protective effects against diarrhea and cough, but that living <0.8 km from dumpsites was associated with greater vomiting and fever based on 1225 interviews with people across four Alaska Native communities (one each from the Northwest, Yukon Interior, Southeast, and the Yukon-Kuskokwim Delta regions) [97]. This survey on health symptoms and solid waste practices also indicated that burning waste near a residence was associated with greater vomiting. They observed that odor complaints and the number of dumpsite visits had dose–response effects for several symptoms, including skin irritation/rash, fever greater than 37.7 °C, earache, headache, upset stomach, eye irritation, headache, and limb numbness, tingling, or weakness.

The Central Council of Tlingit and Haida Tribes & Zender Group (2003) and Zender Group et al. (2003) reports both describe results from the 2000–2001 Central Council of Tlingit and Haida Indian Tribes’ Solid Waste Management (SWM) Survey and Village Health Study, which included 101 Alaska Native communities [23,24]. For a subset of four communities, they also conducted household surveys on health and solid waste practices, interviewing 295 households for a sample size of 1225 individuals; this is the same larger study as reported on in Gilbreath (2004) [97]. They found that residents who regularly visited landfills or dumpsites were 2–3.7 times more likely to report fever, vomiting, stomach pain, headache, numbness, faintness, and ear and eye irritation. Individuals living less than one mile from the dumpsite experienced even greater risk: they were 19 times more likely to report eye irritation and 3–4 times more likely to experience headaches and/faintness. Households who burned their own waste to avoid going to the dumpsite also experienced greater risk of faintness, numbness, rashes, fever, sore throat, and cough.

## 4. Discussion

A more thorough understanding of landfill contaminant transport pathways is necessary to assess environmental and human health threats in rural Alaska, guide future policy changes, and inform regulatory guidelines. However, no comprehensive model exists that synthesizes existing research to integrate our knowledge of how landfill contaminants move through unique Arctic environments. This literature review fills this gap by using 71 studies on landfill contaminants in Arctic environments to produce a conceptual model of how municipal landfills may influence environmental and human health in Arctic environments, such as rural Alaska. This model was based on abundant literature investigating the effects of landfill management and contaminants on the environment, including waste burning, heat generation, carbon release, leachate characterization, and water/sediment contamination. It also highlighted a dearth of research along pathways that are significant for Alaska Native health and culture, particularly those related to erosion, soil contamination, flooding, and subsistence resources. We also found a lack of peer-reviewed literature related to reuse, composting, or backhaul. This absence may be due to limited capacity, the relevance of specific recycling or composting methods to small, remote Arctic communities, or the fact that the material that can feasibly be recycled or composted is a relatively small proportion of the waste stream in these contexts (e.g., [21]). Additionally, most of the literature was greater than 10 years old and may not reflect more recent changes in environmental conditions that could impact landfill contaminant pathways and transport, such as hydrology and permafrost thawing.

Rural Alaska has a distinct context compared to other Arctic locations through a combination of its remoteness, large area, ecological variation, and historical and political context. In this scoping review, we further examined a subset of the studies that took place in Alaska, which are the most relevant for predicting the environmental effects of Class III landfills. We found 33 studies addressing environmental health and six studies addressing human health in Alaska. The research designs of most studies prevented causal inference or generalizability for many measures of environmental and human health. However, synthesizing across studies, we found evidence that landfills in Alaska produce multiple contaminants, particularly heavy metals such as manganese, iron, barium, arsenic, and aluminum [18,81,82,83,85], that can leach into surface and groundwater. In most cases, contaminant concentrations fell to background levels near the landfills; however, not all studies assessed the geographic spread of contaminants via leachate. Based on this work, we suggest that future research includes leachate sampling within Class III landfills and at least 500 feet beyond the impacted zone, an area that includes active or historic waste disposal, significantly disturbed ground, or visibly impacted plant health or diversity. This work should also prioritize investigation of subsistence resource contamination near the landfill to ensure that individuals are not consuming plant material with bioaccumulated contaminants in quantities above human health guidelines. Research on these topics will be most effective if communities are consulted and the research design considers site-specific geological and environmental factors.

Additionally, the literature provided strong evidence that microbes are transported out of landfills, particularly in communities that must use honeybuckets (containers or dry toilets for human waste) due to limited waste infrastructure. Fecal contaminant transport was greater during snowmelt in the spring, suggesting that reductions in comingled waste may have the greatest benefits for human health during wet and warm seasons. Two studies suggested that landfills may affect the health of wildlife through bear or gull consumption of garbage, with potential adverse effects on animal nutrition and pathogen load [75,76]. As gulls are common in Alaska, contaminated feces deposited near homes could lead to AMR *E. coli* exposure, particularly given the evidence in Ahlstrom et al. (2018) and Ahlstrom et al. (2019) that gulls acquire AMR *E. coli* at landfills and can transport it to other locations [73,74]. Further, many households use rainwater catchments to collect rainwater from household roofs [103], suggesting that contaminated gull feces deposited on roofs could also affect water quality. These studies provide preliminary characterization of microbial spread from landfills. However, this work also highlights a critical need for more research on the scope of microbial spread from Class III landfills. Future work should continue to assess microbial transport pathways in communities with honeybuckets. Additionally, no research has assessed the spread of fecal coliforms and other microbes in communities with sewage infrastructure, although microbial spread may be relevant because of diaper disposal into the landfill. Lastly, future work should assess potential impact on rainwater catchment water quality as well as the health of plants and animals that are known to live or feed in the impacted zone.

Heat generation studies provided strong comparative evidence regarding the dynamics of heat generation from solid waste that can affect microbial presence and ground stability [61,63,64,65,98], with implications for landfill contaminant transport. Ongoing and predicted increases in average daily temperatures and increased precipitation may interact additively or even synergistically with heat from solid waste to accelerate permafrost thawing in the future. Permafrost thawing around landfills can also contribute to ground settlement, fence collapse, water ponding, and other issues that increase landfill maintenance costs and create additional challenges for adhering to best practices. As a result, permafrost thaw, coastal and riverbank erosion, and increased precipitation may drive flooding that has the potential to transport leachate offsite via greater surface and groundwater interactions, threatening human and environmental health. However, these insights came from studies of a Class I landfill. The heat generation dynamics of a Class I landfill, of which there are only nine in the state of Alaska, are unlikely to apply to Class III landfills. Future research is needed to investigate the heat dynamics of smaller, unlined landfills without leachate collection systems, with specific attention to the factors that may contribute to greater environmental contamination, including permafrost thawing and leachate transport.

The studies identified in this review were conducted in a scientifically rigorous manner, and each one advances our knowledge of landfill contaminants. However, the research designs of most studies limit their potential for causal inference. For example, among the three studies of soil quality, two used indirect measures to assume contaminant sources or concentration levels, and one focused primarily on military waste landfills rather than municipal landfills. Therefore, this work demonstrates that solid waste can interfere with soil quality. However, more research is needed to assess how chemical and biological processes influence contaminant transport and the effects of exposure. Further, contamination is heterogeneous both within and across landfills, and landfills in Southeast Alaska may create different challenges than other Arctic landfills due to variation in waste streams, precipitation, and climate. Within each landfill, contaminant release will be highly seasonal and dependent on environmental conditions, human activity, and landfill management practices.

Few studies investigated infant or adult health, but those available indicate adverse human health outcomes associated with solid waste exposure, particularly low birth weight, intrauterine growth restriction, and self-reported health symptoms. However, exposures were measured indirectly in all human health studies. For example, exposure was assessed through the presence of low, intermediate, or high hazard dumpsites in the community of the infant’s mother in Gilbreath (2004) [97]. The mother’s exposure and any biological effects would likely be influenced by multiple factors, such as anthropometrics, distance of the residence to the landfill, time spent in the landfill, and the unique characteristics of each landfill, among others. Birth record information is also prone to biases and may not accurately categorize each infant’s health information.

Lastly, one major limitation of this search was the lack of gray literature identified through our methods. Much of the research on solid waste management in Alaska is conducted by non-profit organizations or for-profit companies, and their findings are not always available in the peer-reviewed literature or in public databases. Due to the scope and methods of this review, we were unable to include research findings from gray literature not stored in public databases; however, that work would likely further refine our conceptual model by adding additional pathways for contaminant transport.

Future research should more precisely investigate exposure pathways and measure individual-level exposures, particularly exposure to specific contaminants known to be risk factors for adverse human health (i.e., reproductive outcomes). For example, future projects could leverage research designs with greater causal inference potential and use individual-level measures of exposure consequences. Other areas of interest include investigating contaminants of emerging concern, such as pharmaceuticals and personal care products [104], and a greater consideration of the effects of contaminants on a variety of cultural resources. For example, Mutter (2014) demonstrated that contaminants of emerging concern, such as phthalates, are likely also present in leachate [18]. N-(1,3-dimethylbutyl)-N′-phenyl-p-phenylenediamine-quinone (6PPD-quinone) is another emerging contaminant of concern. 6PPD is a toxic transformation product of tire rubber that can be released into the environment through tire wear. The chemical compound is known to exhibit neurotoxicity and other adverse effects on aquatic and terrestrial organisms and is highly relevant for Alaska Native communities due to its impacts on salmon [105], an important traditional food, and the common occurrence of tires in the solid waste stream [106].

Existing water treatment methods in rural Alaska lack tertiary mechanisms for removing many of these emerging contaminants; stricter regulations, stronger infrastructural support, and improved monitoring strategies are needed to help remove many of these potentially hazardous contaminants from rural Alaska wastewater, such as those identified in Mutter et al. (2014) [18]. Additionally, Gilbreath (2004) calls for greater research to assess how exposure concerns influence subsistence practices, including changes in location, consumption, and substitutions [97]. Given the large seasonal variation in daily activities in rural Alaska, research should also consider how exposure may vary across the year. Lastly, given the potential of human fecal exposure when honeybucket and solid waste are disposed of in the same location, more work is needed to investigate fecal transfer and exposure in honeybucket and non-honeybucket communities, as well as broader questions of how individual or community behavior affect landfill contaminant transport.

Given the unique context of rural Alaska, future work would likely benefit from more thoroughly incorporating community members’ perspectives into the research design. Researchers should also consider partnering with the local non-profits that have extensive solid waste experience and are already working to assist communities and increase their capacity. For many research projects in the Arctic region, local people or organizations can provide insights into environmental processes that are beyond the expertise and experience of outside researchers, improving the rigor of the science and leading to results that more effectively meet the community’s needs and priorities. Doing so will require greater education for researchers to avoid “parachute research,” also known as parasitic or mosquito science. This type of research is an extractive practice whereby researchers travel to a different region, country, or culture (typically low resource settings or countries) to collect data and samples, without sharing the results, acknowledging the importance of the local infrastructure and expertise, or cultivating long-term, equitable, mutually beneficial partnerships [107,108,109]. Research teams may also consider using methods where community partners can formally lend their local expertise, such as through community-based participatory research or applying a co-production of knowledge framework [110]. Similarly, researchers have called for community-engaged research with cultural humility as a foundational value [107], conducting research “in a good way” [111], and have recommended a relational science model that supports Indigenous rights and reconciliation [112].

### Unique Risks for Alaska Native Communities

Addressing landfill contaminant threats and exploring pathways to mitigate these risks directly is especially important for Alaska Native communities, given their close connection to the natural environment and their reliance on natural resources that support their way of life. Although Alaska Native traditions and practices vary greatly across cultural groups, they are universally tied to aspects of the local natural landscape that are key to identity, religion, and way of life. Therefore, injury to the Alaskan natural environment has unique and disproportionate effects for Alaska Native peoples. The loss of culturally essential practices is also linked to numerous adverse mental and physical health outcomes, such as depression and cardiovascular disease [113,114,115]. Additionally, subsistence resources, such as seals, salmon, and berries, remain a significant part of the diet for many Alaska Native peoples [116]. Compared with processed foods imported to the communities, which often have excess packaging that substantially increases landfill waste [117], traditional foods are more nutritious and associated with greater diet quality, lower lipid levels, lower blood pressure, lower glucose levels, and lower adiposity [118,119,120]. However, these subsistence resources can be tainted by contaminants from landfills, making landfill contaminants both an environmental and public health issue [7,8,9,10]. Further, increased health risks from contaminant exposure can contribute to early mortality [121]. Given the importance of elders in Alaska Native communities, the early loss of an elder has a significant cultural impact on the community [95].

Remote Alaska communities, which are predominantly composed of Alaska Native people, also rely on local resources. Discussions with non-profit groups have highlighted the large role of the local, including lifeways (e.g., subsistence activities) and infrastructure (e.g., unpaved roads and boardwalks to landfills) that place Alaska Native peoples at much greater chronic exposure risk relative to individuals in less remote communities. Therefore, contaminants in local drinking water, food, and landfill may affect Alaska Native communities more than non-Native communities because residents experience prolonged local exposure rather than more “incidental” exposure to contaminants that occurs in other populations [122]. Additionally, landfill workers may be at heightened risk due to occupational exposure. These relationships were not explored in the peer-reviewed literature that we encountered; additional research is needed to assess what is known in the gray literature and more thoroughly investigate the contributions of cultural and contextual factors to risk or mitigation of exposure in rural Alaska communities.

The environmental and human health risks of landfill contaminants are not limited to their public or ecosystem health consequences but also have the potential to harm Alaska Native culture and subsistence food systems. Therefore, among the future research directions identified above, we propose that subsequent work prioritize research with the greatest implications for assessing the risk to subsistence resources given their importance for Alaska Native culture and health. Specifically, we recommend that future research assess the potential effects of leachate and bacterial contamination on soil, water, plants, wildlife, and human health, although communities themselves should determine the final priorities. This work should go beyond merely characterizing contamination to develop and test affordable and practical interventions to reduce this contamination.

## 5. Conclusions

In this scoping review, we assessed current knowledge of the relationship between landfill contaminants to both environmental and human health in the Arctic, in order to create a conceptual model of contaminant transport pathways. Among the 71 studies that addressed this objective, we found that most research focused on water/sediment contamination, heat generation, carbon release, waste burning, pathogen exposure risk, and leachate characterization. Little work assessed air quality, human health, animal health, or transport pathways related to erosion, soil contamination, flooding, or subsistence resources. Secondarily, we investigated the relationship between landfill contaminants and environmental or human health in Alaska. As a result, this scoping review highlights the known consequences of landfill contaminants in rural Alaska that have importance for Alaska Native communities. It also demonstrates the limitations and gaps of prior research in the peer-reviewed literature, both of which must be further addressed to prevent continued environmental harm for Alaska Native communities.

Among the 39 identified studies that focused on Alaska, we found evidence that Class III landfills in Alaska produce contaminants that can leach into groundwater, including heavy metals. The studies also supported the relationship between honeybucket use and fecal microbe migration beyond landfill site boundaries in communities where human waste and solid waste are commingled, particularly when snowmelt occurs in the spring. In communities that use honeybuckets, fecal bacteria are transferred most efficiently to soft, moist surfaces, which may increasingly pose a risk to human health if warmer and wetter weather trends in Alaska continue. Heat generation studies suggest that decomposition influences temperature and moisture content of landfills, although work specific to Class III landfills is still needed. Very little research focused on human health; however, existing work suggests that solid waste may be related to adverse birth outcomes. Specifically, one study found that infants in communities with high hazard dumpsites were more than four times more likely to have congenital anomalies classified as “other” than those in communities with low or intermediate hazard dumpsites. Additionally, living in close proximity to the landfill was associated with a greater frequency of vomiting and fever. No research investigated air quality measures, such as particulate matter (PM) 2.5, PM10, or methane emissions.

The small number of studies identified in this review strongly indicate that Class III landfills in Alaska have the potential to influence environmental and human health. However, we also identified numerous research gaps. We call for greater research on individual-level exposure to landfill contaminants, more robust characterization of contaminant transport pathways, and the use of research designs that facilitate causal inference and incorporate community members’ local expertise and knowledge. We also recommend that future research address specific research gaps noted above, prioritizing those that provide the data most relevant for subsistence activities and human health, such as studies to investigate the relationship of specific contaminants of interest with soil, plant, wildlife, and human health. Further research should also assess the effects of microbial transfer from commingled human waste and solid waste and develop solutions that reduce contamination using feasible methods. Such a research approach would provide data that could be used to characterize the scope and nature of the environmental health issues involved. This data would allow for an accurate cost benefit analysis between greater investment in rural Alaska landfill infrastructure compared to the environmental and public health costs of landfill contaminant exposure, which may include adverse effects on mental and physical health, reduced work productivity, loss of environmental integrity, cultural impact, social behavioral impacts, emigration, governmental liability and more. Such future research would be aligned with the government’s commitment to equal treatment of individuals and communities regardless of race, ethnicity, or gender and likely drive potential funding to ameliorate the problem, with the community tangibly benefiting in the process. Given the large potential for landfill contaminants to adversely affect environmental and human health in combination with the existing dearth of knowledge on these topics, extensive research is needed to continue to assess landfill contaminant transport, exposure risks and the unique challenges of solid waste management in rural Alaska.

## Data Availability

No new data were created or analyzed in this study. Data sharing is not applicable to this article.

## Supplementary Materials

The following supporting information can be downloaded at: https://www.mdpi.com/article/10.3390/ijerph23010045/s1, File S1: Preferred Reporting Items for Systematic reviews and Meta-Analyses extension for Scoping Reviews (PRISMA-ScR) Checklist.

## Appendix A

**Table A1:** List of databases and search strings for Alaska Waste and Health Impacts Literature Search (October 2021).

Database	Search String	Date Run & Results
PubMed	(((“Open landfill”[tw] OR “Dump sites”[tw] OR “Dumpsites”[tw] OR “Open Dump”[tw] OR “Unlined landfill”[tw] OR “Class III landfill”[tw] OR “E-waste”[tw] OR “electronic waste”[tw] OR “Solid waste”[tw] OR “Solid waste disposal”[tw] OR “Household hazardous waste”[tw] OR “Hazardous waste”[tw] OR “Human waste”[tw] OR “Honeybucket”[tw] OR “Construction waste disposal”[tw] OR “demolition waste disposal”[tw] OR “Ash”[tw] OR “Mixed waste”[tw] OR “Leachate”[tw] OR “Lagoon”[tw] OR “holding pond”[tw]) AND (“Alaska”[tw] OR “Arctic”[tw] OR “Rural”[tw] OR “Indigenous”[tw] OR “Cold region”[tw] OR “Alaska Native Village”[tw] OR “Alaska Tribe *”[tw] OR “Alaska Tribal”[tw] OR “Indian land *”[tw])) AND 1980:3000 [dp])	10 December 2021 788 results HERO Import Batch ID: 45090
Web of Science Core Collection	(((TS = “Open landfill” OR TS = “Dump sites” OR TS = “Dumpsites” OR TS = “Open Dump” OR TS = “Unlined landfill” OR TS = “Class III landfill” OR TS = “E-waste, electronic waste” OR TS = “Solid waste” OR TS = “Solid waste disposal” OR TS = “Household hazardous waste” OR TS = “Hazardous waste” OR TS = “Human waste” OR TS = “Honeybucket” OR TS = “Construction waste disposal” OR TS = “demolition waste disposal” OR TS = “Ash” OR TS = “Mixed waste” OR TS = “Leachate” OR TS = “Lagoon” OR TS = “holding pond”) AND (TS = “Alaska” OR TS = “Arctic” OR TS = “Rural” OR TS = “Indigenous” OR TS = “Cold region” OR TS = “Alaska Native Village” OR TS = “Alaska Tribe *” OR TS = “Alaska Tribal” OR TS = “Indian land *”)) AND (PY = 1980–2022))	10 December 2021 2888 results HERO Import Batch ID: 45091

Notes: There are 2887 records tagged for WOS on the project page because there was one duplicate record with two separate WOSIDs within the imported results.

## Appendix B

Instructions for Alaska landfill screening review:

1. Make include/exclude decision.

Include if paper explicitly deals with one of more of these topic areas: (1) waste disposal in landfills or open dumps in rural areas, especially in arctic/subarctic climates or on indigenous lands; (2) health or environmental effects related to these types of waste disposal, including changes in water, soil, or air quality or biota; (3) management or remediation methods related to these types of waste disposal.

Exclude if paper does not deal with the topics detailed above.

2. For included papers, apply the following tags where relevant (individual tags and comment fields are indicated in bold).
  - **Abstract—comment**: note if abstract is missing; if abstract is missing, assign tags below based on title, where possible
  - LOCATION: geographic area addressed in paper
    ◦ **Alaska**
    ◦ **Other arctic/subarctic regions**
      ▪ **North America**
      ▪ **Europe**
      ▪ **Asia**
      ▪ **Other**
    ◦ **Non-arctic/subarctic regions**
      ▪ **US**
      ▪ **Non-US**
      ▪ **Not specified**
    ◦ **Indigenous land**
    ◦ **Non-indigenous rural land**
    ◦ **Not specified:** location is not stated or clear based on title/abstract
  - **Landfill/waste disposal type—comment**: note what type of landfill or waste disposal is addressed in the paper (e.g., lined or unlined, any waste burning, etc.), or if landfill or waste disposal type is not specified
  - WASTE TYPE: types of waste addressed in paper
    ◦ **Not specified**
    ◦ **Human waste:** e.g., biosolids
    ◦ **Household waste:** e.g., domestic wastes
    ◦ **Hazardous waste**
    ◦ **Construction/demolition waste**
    ◦ **Electronic waste**
  - **Waste type—comment**: note any information about specific types of waste addressed in paper, particularly if none of the above tags seem appropriate
  - TOPICS: specific topic areas addressed in paper
    ◦ **Air quality**
    ◦ **Water quality**
    ◦ **Soil quality**
    ◦ **Human health effects**
    ◦ **Other environmental effects**
    ◦ **Management/remediation**
  - **Topics—comment**: briefly note any information about topics of interest addressed in paper, particularly if none of the above tags seem appropriate
